# Long-term progressive deterioration of visual function after papilledema improved by embolization of a dural arteriovenous fistula in the sigmoid sinus: a case report

**DOI:** 10.1186/1752-1947-8-392

**Published:** 2014-11-28

**Authors:** Masahiro Zako, Kazuhiro Murata, Takashi Inukai, Muneyoshi Yasuda, Masayoshi Iwaki

**Affiliations:** 17 Department of Ophthalmology, Aichi Medical University, Nagakute, Aichi, 480-1195 Japan; 18 Department of Neurological Surgery, Aichi Medical University, Nagakute, Aichi, Japan

**Keywords:** Dural arteriovenous fistula, Papilledema, Sigmoid sinus, Visual function

## Abstract

**Introduction:**

It is generally believed that people affected by papilledema will not have progressive damage to their eyesight if they receive adequate medical care to treat the underlying cause of the papilledema. We present a case that appears to contradict this widely accepted belief.

**Case presentation:**

A 53-year-old Japanese man with tinnitus visited our hospital. His initial best-corrected visual acuity in either eye was not impaired, although they both exhibited papilledema. Magnetic resonance imaging did not reveal a mass or hemorrhagic lesion in our patient’s brain. Nevertheless, his best-corrected visual acuity gradually deteriorated over the following three months. Angiography demonstrated a dural arteriovenous fistula in his sigmoid sinus. After embolization therapy, the papilledema improved in both eyes. However, over the subsequent four years, his best-corrected visual acuity progressively deteriorated due to an unknown cause, despite the successful embolization of the dural arteriovenous fistula.

**Conclusion:**

There may be delayed onset of an unknown pathophysiology in the visual system after treatment for the underlying cause of papilledema, implying an uncertain visual prognosis for patients with this condition.

## Introduction

Papilledema, also known as choked disc, is an edema of the optic nerve head that is caused by elevated intracranial pressure [1, 2]. This condition is usually bilateral and results from a cerebral tumor, cerebral abscess, meningitis, encephalitis, subarachnoid hemorrhage, head injury, hydrocephalus, or many other local or systemic pathological conditions. In the early stages, the patient may have a headache, but visual acuity may not be affected. In advanced stages, the papilledema may progress to an enlargement of the blind spot in visual field measurements, blurred vision, visual obscurations, or even total loss of vision. The timeline of visual impairment associated with papilledema is highly variable; major deficits can arise within weeks in severe cases, but more typically they arise over several months [3]. The risk of impaired visual acuity increases as the duration of the edema increases [4], although the critical duration has not been determined. However, a person affected by papilledema is generally not expected to have progressive damage to their eyesight if they receive adequate medical care for the underlying cause of the papilledema.

We present the case report of a patient with bilateral papilledema, which was caused by a dural arteriovenous fistula (DAVF) in his sigmoid sinus. DAVFs are abnormal arteriovenous connections within the dura mater that involve a dural sinus and/or cortical veins. The most common location for DAVFs is the transverse or sigmoid sinus [5].

After administering the appropriate transarterial and transvenous embolization therapy, our patient’s bilateral papilledema improved considerably. However, his visual acuity then progressively deteriorated, with consequent disc paling, although he did not exhibit thinning of the retina. Our experience with this patient suggests that there may be delayed onset of an unknown pathophysiology in the visual system after treatment for the underlying cause of papilledema, implying an uncertain prognosis for patients with this condition.

## Case presentation

**Figure 1:**
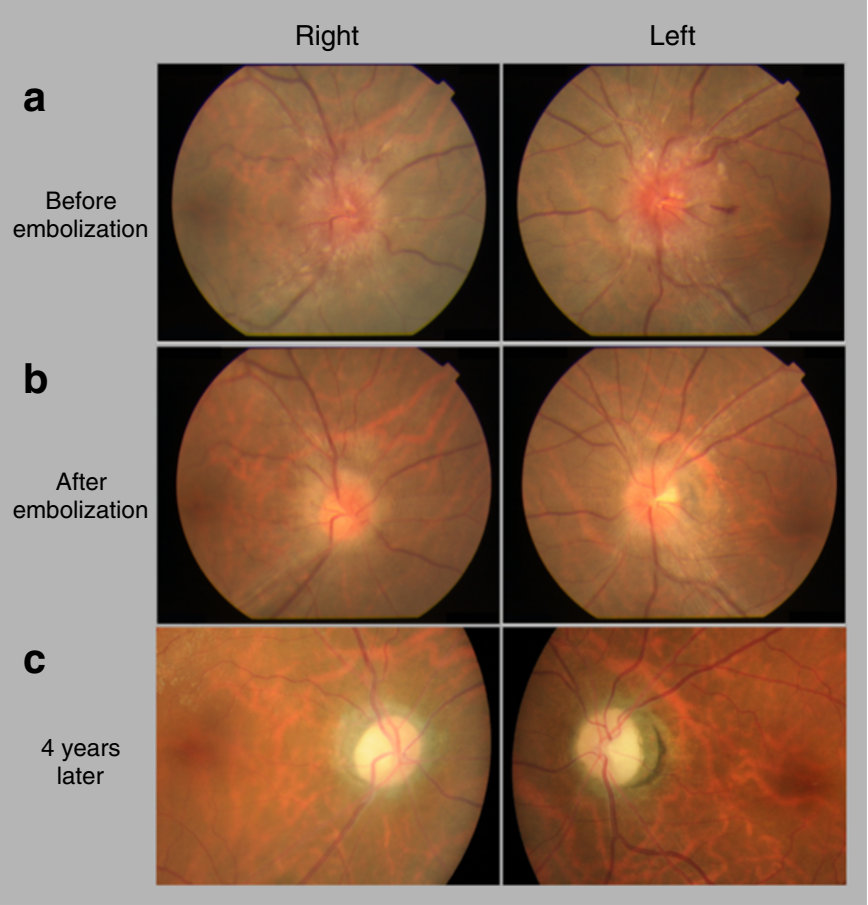
**Photographs showing optic discs before and after treatment. (a)** Optic disc on the first visit. **(b)** Optic disc three months after transarterial and transvenous embolization therapy. **(c)** Optic disc four years after embolization therapy.

A 53-year-old Japanese man presented to our hospital complaining of tinnitus over a period of three weeks. He also noted slightly abnormal “fuzzy” vision in the previous two weeks. An otorhinologist was consulted to rule out Vogt–Koyanagi–Harada disease.An ocular evaluation revealed a best-corrected visual acuity (BCVA) of 1.2 in his right eye and 1.0 in his left eye. His intraocular pressure was 14mmHg in his right eye and 16mmHg in his left eye. The anterior segment was normal in both eyes. However, a fundus evaluation revealed a significant papilledema in each eye (Figure 1).

Brain magnetic resonance imaging (MRI) demonstrated the absence of an intracranial mass or hemorrhagic lesion. Our patient did not experience hypertension or uveitis; at that point, he had experienced no loss of BCVA. We did not measure our patient’s intracranial pressure because he did not approve the procedure. Because he did not demand further examination, we held follow-up appointments with our patient every two weeks.

Our patient had been taking aripiprazole for depression for over seven years. He was also diagnosed with mild cognitive impairment, also known as incipient dementia, for which he was not receiving treatment.

**Figure 2:**
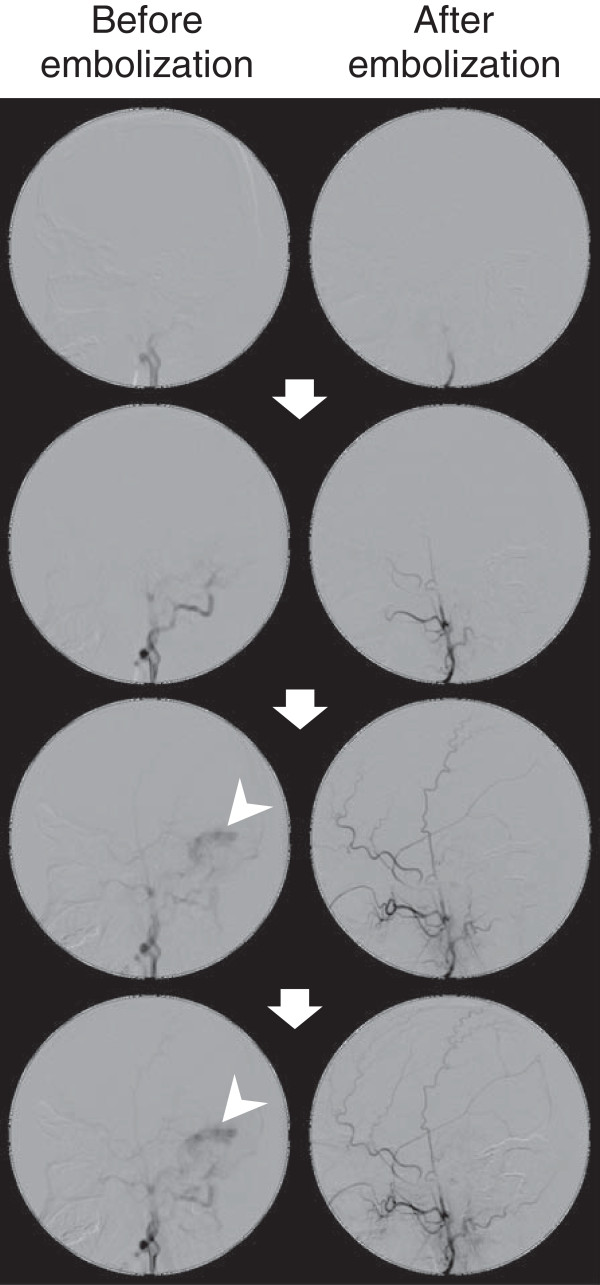
**Angiography before and after embolization therapy.** Right external carotid angiography (lateral view) demonstrates blood flow into the sigmoid sinus venosus (arrowheads) through the dural arteriovenous fistula. Paired photographs show images of the same phases after embolization therapy. Arrows indicate the time course, from top (early) to bottom (late).

**Figure 3:**
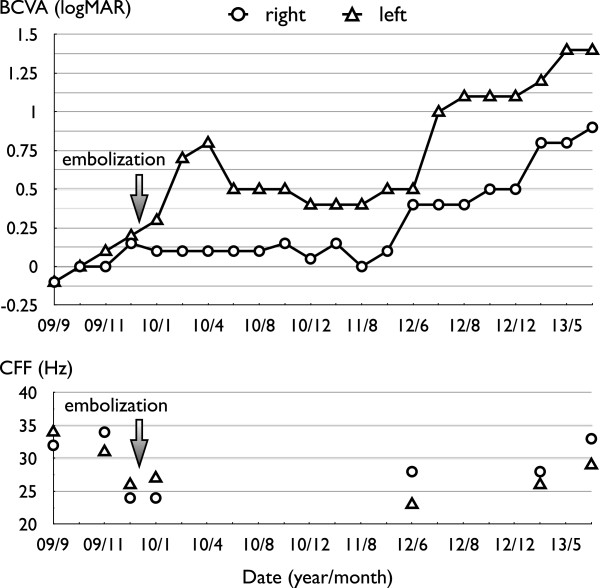
**Best-corrected visual acuity and critical fusion frequency of the patient.** BCVA, best-corrected visual acuity; CFF, critical fusion frequency.

**Figure 4:**
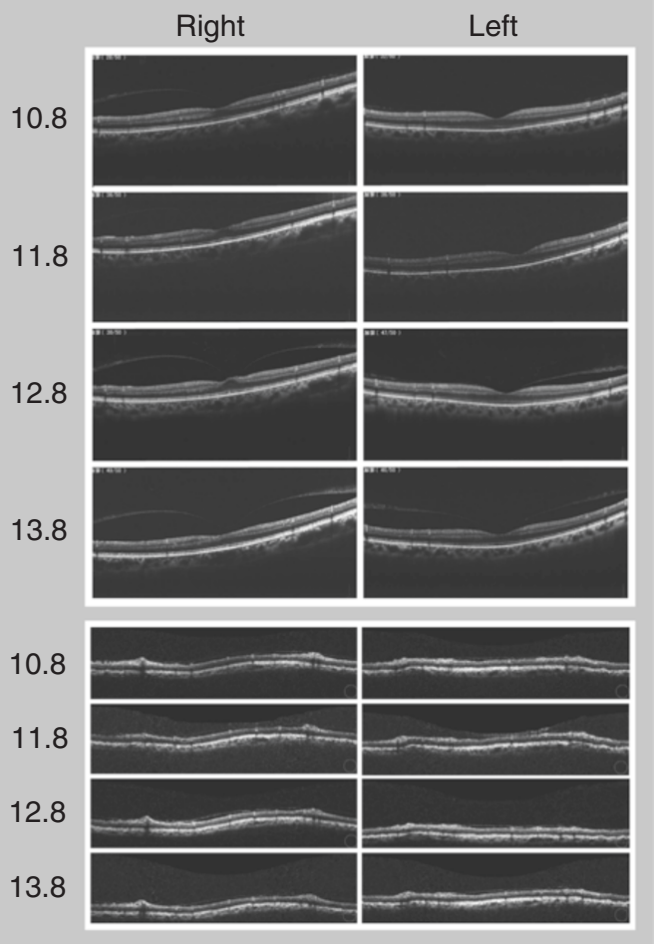
**Images of macula and retina around the disc, analyzed by optical coherence tomography over the last three years.**

**Figure 5:**
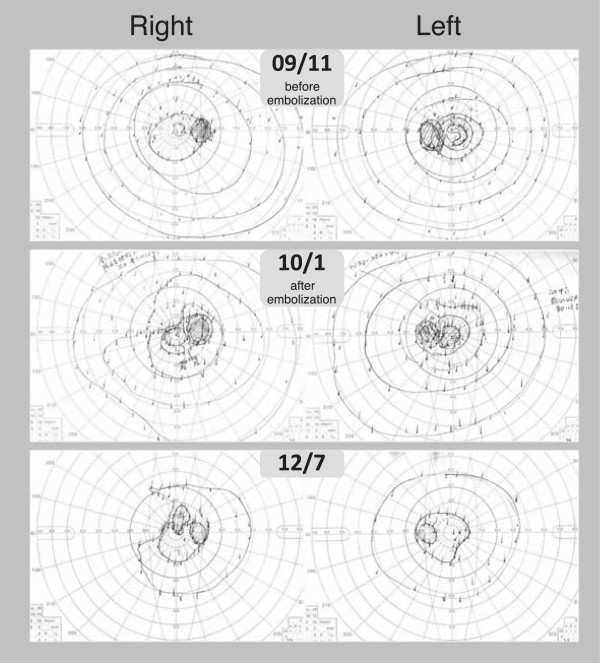
**Goldmann kinetic visual field, obtained before and after transarterial and transvenous embolization therapy.**

Ten weeks after his first visit to our hospital, our patient began to experience sporadic marked blurred vision and transient day blindness. His BCVA had deteriorated moderately to 0.9 in his right eye and 0.8 in his left eye. A neurosurgeon detected a DAVF in his sigmoid sinus on angiography. The fistula was classified as type I, according to Borden classification, and type IIa, according to Cognard classification.Transarterial and transvenous embolization was successfully performed to treat the causative DAVF. Three weeks after therapy, the papilledema had significantly improved (Figure 1) but the BCVA in our patient’s right and left eyes was 0.8 and 0.3, respectively. His tinnitus disappeared by one week after embolization therapy. The angiography results before and after embolization are presented in Figure 2.We followed this patient for four years. Regular ocular examinations demonstrated the absence of papilledema recurrence. However, the patient’s BCVA continued to deteriorate progressively (Figure 3), with concurrent complete disc paling (Figure 1) but without thinning of the foveal retina or retina around the disc, as determined by optical coherence tomography (OCT) (Figure 4).A mild vitreomacular adhesion in his right eye was revealed by OCT with SDOCT RS-3000 (Nidek Co., Ltd, Aichi, Japan) and Nidek Advanced Vision Information System EX version 1.3.0.3 (Nidek Co., Ltd) (Figure 4). Our patient did not notice metamorphopsia, and he did not request pars plana vitrectomy. Goldmann perimetry showed progressive narrowing of the visual field in both eyes after embolization (Figure 5).

Four years after embolization, our patient’s BCVA was 0.15 in his right eye and 0.04 in his left eye. His intraocular pressure was within normal range in both eyes at all examinations (data not shown). Our patient’s intracranial condition was examined by MRI and magnetic resonance angiogram every six months, but he never had a recurrence of the right DAVF in the sigmoid sinus, nor were there any other abnormal findings.

## Discussion

Patients with papilledema are generally not expected to have progressive damage to their eyesight if they receive adequate medical care to treat the underlying cause of the papilledema. However, the prognosis for visual function can not be determined simply by measuring the extent of damage before treatment. As shown in this case, visual function can continue to deteriorate despite an initial improvement after effective surgery.

To the best of our knowledge, our case report is the first in the literature to describe the progressive deterioration of visual function after appropriate treatment in a patient with DAVF. We speculate that there may have been delayed onset of an unknown pathophysiology in his visual system after treatment for the underlying cause of papilledema, implying an uncertain prognosis with respect to the disease.

It is unclear what caused the initially improved visual function to deteriorate in the few years following treatment. Papilledema can have a number of effects on the visual system, the most severe of which is nerve fiber dysfunction from swelling, with subsequent and progressive loss of the retinal nerve fibers and optic atrophy [6]. Although the definitive pathogenesis of papilledema in response to increased intracranial pressure remains unclear, the prevailing theory is that papilledema is primarily a mechanical nonvascular phenomenon [7, 8]. According to this theory, optic disc swelling causes axon compression, which results in hypoxia, ischemia, gliosis, and, finally, optic atrophy due to delayed death or degeneration of neuronal or retinal cells [6, 9]. Another possibility for the mechanism underlying the delayed neurodegeneration is apoptosis. Optic nerve lamina cribrosa cells that are exposed to hypoxic stress may be involved in apoptosis and neurogenesis [10].

A technique was developed in monkeys to produce papilledema by inflating intracranial balloons [11]. With this method, papilledema did not develop in optic nerves subjected to procedures that resulted in total optic atrophy prior to the elevation of intracranial pressure [12]. Thus, in following up the cause for papilledema in a patient with total optic atrophy, we should use not only fundoscopy but also other appropriate examinations such as intracranial pressure measurement, MRI, and magnetic resonance angiography (MRA).

No remarkable progressive abnormal retinal atrophy was detected by OCT in our case. A central scotoma was detected just after embolization in our patient’s left eye, and another scotoma was detected 2.5 years after therapy in his right eye, suggesting the involvement of optic neuropathy. However, his visual field did not indicate a more specific disease or lesion. The precise mechanism of the delayed-onset optic atrophy remains unclear.

A dural arteriovenous malformation is difficult to recognize with brain computed tomography (CT) or MRI, but it can be well documented by MRA and cerebral angiography [13]. In this case, we spent almost three months obtaining an accurate diagnosis, as initial cranial CT and MRI images did not show any abnormal findings. The DAVF was a cause of the papilledema in this case. The pathogenic theory of papilledema is that it is caused by an increase in cerebral blood volume due to impaired cranial venous outflow, leading to intracranial hypertension [14]. Therefore, if a patient has bilateral papilledema, clinicians should perform MRA or cerebral angiography to rule out intracranial circulatory malformations [15].

If a patient has papilledema plus depression and/or dementia, DAVF should be considered as a cause of these disorders. However, the relationships among DAVF, deteriorated visual function, and symptoms of depression and dementia are not straightforward. DAVF is a cause of depression [16–18], and depression may improve after the DAVF is treated [16]. DAVF is also implicated as a cause of dementia, and dementia may be reversed after treatment for the DAVF [19–25]. Deteriorated visual function itself may elicit depressive symptoms in patients [26–28]. A possible explanation for the lack of significant improvement in our patient’s symptoms of depression may be that his visual function continued to deteriorate.

## Conclusion

We have described our experience with a case of bilateral papilledema, which was induced by a DAVF in the sigmoid sinus and subsequently improved following embolization therapy. However, our patient’s visual function progressively deteriorated after the successful therapy. This delayed onset of unknown pathophysiology in the visual system after treatment for the underlying cause of papilledema implies an uncertain prognosis for the disease.

## Consent

Written informed consent was obtained from the patient for publication of this case report and any accompanying images. A copy of the written consent is available for review by the Editor-in-Chief of this journal.

## Abbreviations

BCVA: Best-corrected visual acuity
CT: Computed tomography
DAVF: Dural arteriovenous fistula
MRA: Magnetic resonance angiography
MRI: Magnetic resonance imaging
OCT: Optical coherence tomography.
